# Long-term follow-up of kidney transplant recipients: comparison of hospitalization rates to the general population

**DOI:** 10.1186/2047-1440-2-15

**Published:** 2013-08-24

**Authors:** Ying Jiang, Paul J Villeneuve, Douglas Schaubel, Yang Mao, Panduranga Rao, Howard Morrison

**Affiliations:** 1 Science Integration Division, Public Health Agency of Canada, 785 Carling Ave., Ottawa, Ontario K1A 0K9, Canada; 2 Institute of Health: Science, Technology and Policy, Carleton University, 1125 Colonel By Drive, Ottawa, Ontario K1S 5B6, Canada; 3 Department of Biostatistics, University of Michigan, 1420 Washington Hts, Ann Arbor, MI 48109-2029, USA; 4 Internal Medicine - Nephrology, University of Michigan, 102 Observatory, Ann Arbor, MI 48109-5725, USA

**Keywords:** Cohort study, Kidney transplantation, Record linkage, Standardized hospitalization ratio

## Abstract

**Background:**

Kidney transplant recipients are recognized as a vulnerable population that is at increased risk of adverse health outcomes. However, there have been few studies that have compared hospital-related morbidity of these patients to the general population, and how this differs with respect to time since transplantation. Such analyses are useful in estimating the health burden in this patient population.

**Methods:**

We assembled a population-based Canadian cohort (excluding Quebec) of 6,116 kidney transplant recipients who underwent transplantation between 1 April 2001 and 31 December 2008. Record linkage was used to identify hospital discharge records of these patients from 1 April 2001 through 31 March 2009. Hospital discharges were tabulated across the main disease chapters of the ICD10, and person-years of follow-up were calculated across age and sex strata. Comparisons of hospital-related morbidity to the general population were made by using a standardized hospitalization ratio (SHR). For those who underwent transplantation in 2004, stratified analyses were performed to explore differences in hospital discharge rates both before and after transplantation.

**Results:**

After excluding hospitalizations due to complications from transplantation, when compared to the general population, transplant recipients were approximately 6.4 (95% CI: 6.3, 6.5) times more likely to be hospitalized during follow-up. The SHRs were highest during the time periods proximate to transplantation, and then decreased to approximately a five-fold increase from 3 years post transplantation onwards. The largest disease-specific excesses were observed with infectious diseases and diseases of the endocrine system. Among those who underwent transplantation in 2004, the SHR decreased from 11.2 to 5.0 in the periods before and after surgery, respectively.

**Conclusions:**

Our results indicate that, even more than 5-years post transplantation, there remains a more than six-fold difference in hospitalization rates relative to the general population. Additional work is needed to confirm these findings, and to develop strategies to reduce long-term morbidity in this patient population.

## Background

In Canada, the prevalence of end-stage renal disease has increased steadily over the past 20 years [1-3]. Kidney transplantation is the preferred treatment among individuals with end-stage renal disease, and improvements in treatment have produced a 1-year survival probability following transplantation that now exceeds 90% [4]. Improved survival implies a substantially increased opportunity to experience adverse health events post transplantation. Kidney transplant recipients have higher incidence rates of cancer, particularly non-Hodgkin’s lymphoma and skin cancer when compared to the general population [5,6]. These recipients are also more likely to be at an increased risk of developing cardiovascular disease [7,8], diabetes mellitus [9,10], fractures [11-13], and infection [14] post transplantation. In addition, some have recently suggested that transplant patients are more likely than the general population to have higher rates of depression or other psychiatric illnesses [15,16].

To date, there have been relatively few studies that have evaluated the long-term hospitalization patterns among kidney transplant patients. Those that have were typically single-center studies that have been focused on specific diseases and were based on relatively small numbers of transplant patients [9,13,15]. Hospitalization admissions patterns have previously been evaluated using data from the Dialysis Outcomes and Practice Patterns Study (DOPPS) that examined hospital admissions among dialysis patients. Unfortunately these analyses excluded transplant patients [17]. Another study [18] based on US transplant patients was more focused on statistical methods and did not provide comparisons to the general population. In our view, there is insufficient information to adequately characterize the long-term hospitalization burden in the post-transplant renal recipient.

This study aims to address two important research questions that remain unanswered. First, to what extent does transplantation reduce hospital-related morbidity among kidney transplant recipients post transplantation; and second, does the increased need for hospitalization among transplant recipients change with increasing time since transplantation? We used two population-based administrative datasets to create a longitudinal dataset that allows these research questions to be addressed.

## Methods

### Study population

Our study cohort was created by abstracting data from a national information system for renal and extra-renal organ failure and transplantation in Canada, the Canadian Organ Replacement Registry (CORR), which is maintained by the Canadian Institute for Health Information (CIHI). Data are provided to the CORR from all 27 kidney transplant programs across Canada. It has been estimated that the CORR covers approximate 99% of all transplant patients in Canada [19]. Patients included in this analysis consisted of those in the CORR database who underwent renal transplantation between 1 April 2001 and 31 December 2008. We restricted analyses to records that represented an individual’s first transplantation. Patients who underwent more than one kidney transplantation were excluded from analysis. Individual-level variables extracted from the CORR included date of birth, gender, province of residence, and vital status.

### Ascertainment of hospital admissions

The hospitalization experience of the cohort members was determined by linking the personal identifiable data from the CORR to information from the Canadian Discharge Abstract Database (DAD) [20]. The DAD is a population-based database that contains information on all separations from acute care institutions, including discharges, deaths, sign-outs, and transfers (excluding Quebec). It has been estimated that the DAD captures >95% of all hospitalization in Canada (excluding Quebec).

The DAD and CORR record linkage process generated an analysis dataset for the time period between 1 April 2001 and 31 March 2009 identifying hospital discharges among the renal transplant patients. The Most Responsible Diagnosis was used at the time of hospital discharge to classify morbidity among the study population. Hospital admissions were classified based on International Classification of Diseases-10 version (ICD10) chapter definitions [21]. Since DAD does not capture information in Quebec, all records from Quebec in CORR were also excluded for the linkage and analysis.

Since hospitalization during the first couple of months after renal transplant likely reflects morbidity related to the transplant procedure itself, the follow-up interval within the first 60 days after the procedure was examined separately. Initially, a total of 6,116 renal transplant recipients who underwent transplantation between 1 April 2001 and 31 December 2008 were identified. Patients who ended the follow-up within the first 60 days after transplant (*n*=37) were analyzed only for the short-term follow-up in the study. In summary, our risk estimates for medium- and long-term hospital utilization were based on 6,079 renal transplant patients. All transplant patients were followed up until either death (between 1 April 2001 and 31 March 2009) or the end of the study (31 March 2009).

### Statistical analysis

Person-years of follow-up and the number of hospitalizations were tabulated across strata defined by: 5-year age-group, sex (male, female), length of follow-up (<60 days, 60 days to <3 years, 3 years to <5 years, and 5 years and over), and calendar year (2001 to 2008). The DATAB module in the Epicure software program was used to tabulate these person-years [22]. Hospital discharge rates for the Canadian general population exclude Quebec were also obtained from the DAD, and were computed across the same age-group and sex strata.

Hospital discharge rates for the kidney transplant cohort were compared to those of the general population using the Standardized Hospitalization Ratio (SHR). The SHR represents the ratio of observed-to-expected numbers of hospital admissions, with the ‘expected’ reflecting the number of hospitalizations that would be expected in the transplant cohort if it had the same age-sex specific hospital discharge rates as the general population. The 95% confidence intervals (CIs) for the SHRs were constructed using the normal approximation [23].

Disease categories for complications of procedures/ transplanted organs rejection, follow-up examination and adjustment, and other supplementary classifications related to transplant procedure (ICD-10 codes: S01-T99, V01-Y98, and Z00-Z99) were investigated separately and not included in the SHRs comparison. Statistical significance was determined based on a two-tailed alpha of 5%. To adjust for differences in the sex and length of follow-up distribution, the stratified SHRs were also calculated. Analyses were repeated for each of the following main disease categories comprising the ICD-10. The proportions of hospital admission on major disease category were calculated by length of follow-up and compared to those in the general population.

To evaluate whether transplantation reduced morbidity, we conducted a period-rates comparison among individuals having sufficient follow-up time both before and after surgery. Specifically, among kidney transplant recipients who underwent transplantation between 1 April 2004 and 31 March 2005 we compared the SHRs in the pre-transplant (1 April 2001 to 31 March 2003) and post-transplant periods (1 April 2006 to 31 March 2008).

## Results and discussion

### Results

#### ***Descriptive statistics***

The basic statistics for the study cohort are provided in Table 1. The cohort consisted of 6,116 renal failure patients who underwent transplantation between 1 April 2001 and 31 December 2008. The mean age of the recipients was 47.2 years, and the 25th and 75th age percentiles were 37.2 and 58.8, respectively. Approximately 62% of the cohort was male. In total, 22,619.3 person-years were accrued between the date of transplantation and the end of follow-up. Approximately 8% of the cohort (*n*=482) was deceased at the end of the follow-up interval, of which 37 patients died between within the first 60 days post transplant. In the study, 6,079 patients were followed-up for post-transplant hospital admissions commencing 60 days after the transplant.

**Table 1:** Descriptive characteristics of kidney transplant recipients in the Canadian Organ Replacement Registry (excluding Quebec) undergoing transplantation between April 2001 and December 2008

**Characteristic**	**Patients ( *n *)**	**%**
Sex		
Male	3,779	61.8
Female	2,337	38.2
Age at transplant (years)		
<15	213	3.5
15 to <40	1,600	26.2
40 to <55	2,139	35.0
55 to < 70	1,905	31.1
70 and over	259	4.2
Total length of follow-up		
< 60 days^a^	37	0.6
60 days to < 3 years	2,588	42.3
3 years to < 5 years	1,538	25.2
5 years and over	1,953	31.9
Year of renal transplant		
2001	538	8.8
2002	712	11.6
2003	719	11.8
2004	728	11.9
2005	762	12.4
2006	879	14.4
2007	904	14.8
2008	874	14.3
Total (excluding the first 60 days)	6,079	99.4
Total	6,116	100.0

^a^These subjects were excluded in the estimation of hospitalization.

#### ***Hospital admission rates***

A total of 17,431 hospitalizations were observed among the kidney transplant recipients over the follow-up interval. After excluding patients with <60 days of follow-up and hospitalization for transplant procedure related causes, the total number of hospital admissions in this cohort was 14,147. The SHR was 6.4 relative to the Canadian general population, with similar ratios for men and women (male: SHR=6.2; female: SHR=6.5). Among disease categories, SHRs were most elevated for infectious diseases, endocrine disorders, and congenital anomalies. Complications of pregnancy in women was the only condition for which the hospitalization rates were lower among the kidney transplant patients relative to the general population (SHR=0.2) (Table 2).

**Table 2:** **Number of hospitalizations and standardized hospitalization ratios (SHRs) relative to the general population among 6,079 kidney transplant patients, between April 2001 and December 2008, by disease category**^**a**^

**Disease grouping**	**ICD10**^**b**^	**Kidney transplant patients**			
		**Observed**	**Expected**	**SHR**	**95% CI**
All hospitalizations		14,147	1,876.1	7.5	7.4, 7.7
All hospitalizations^c^	Excluding S01-T99, V01-Y98, and Z00-Z99	10,227	1,604.6	6.4	6.3, 6.5
Infectious diseases	A00-B99	853	27.4	31.2	29.1, 33.4
Neoplasms	C00-D48	498	171.2	2.9	2.7, 3.2
Blood disorders	D50-D89	201	16.5	12.2	10.6, 14.0
Endocrine disorders	E00-E99	1,114	50.6	22.0	20.7, 23.3
Mental disorders	F00-F99	80	84.7	0.9	0.7, 1.2
Nervous system and sense organs	G00-G99, H00-H95	483	43.2	11.2	10.2, 12.2
Circulatory	I00-I99	1,078	298.6	3.6	3.4, 3.8
Respiratory	J00-J99	661	123.4	5.4	4.9, 5.8
Digestive	K00-K93	1,480	227.0	6.5	6.2, 6.9
Skin and subcutaneous tissue	L00-L99	165	19.9	8.3	7.1, 9.7
Musculoskeletal	M00-M99	420	128.8	3.3	3.0, 3.6
Genitourinary	N00-N99	2,132	120.2	17.7	17.0, 18.5
Complications of pregnancy	O00-O99	38	158.2	0.2	0.2, 0.3
Congenital anomalies	Q00-Q99	53	2.8	18.9	14.2, 24.8
Symptoms ill defined	R00-R99	968	114.6	8.4	7.9, 9.0

^a^Follow-up interval was from 60 days after the date of their renal transplant to the date of death or until 31 March 2009.

^b^ICD 10, International Classification of Diseases, 10th.

^c^Disease category on complications of procedures/ transplanted organs rejection, follow-up examination and adjustment, and other supplementary classifications related to transplant procedure (ICD-10 codes: S01-T99, V01-Y98, and Z00-Z99) was excluded.

During the first 60 days after the transplant procedure, 60% of hospital admissions were attributable to the procedure itself or to related causes which included complications of procedure, transplanted organs rejection, and follow-up examination and other factors influencing health status (Table 3). This decreased to 30% thereafter. After excluding procedure-related causes, during the first 60 days post transplant, the patient cohort experienced higher standardized hospitalization ratios due to infectious disease, endocrine and genitourinary disease, and lower SHRs for neoplasms and circulatory disease. In the long-term follow-up analysis the proportions of hospital admission of major causes of disease, such as circulatory disease and respiratory disease, tended to return closely to those in the general population (data not shown). There were significant differences on hospital admission rates by province during the follow-up periods. Between 2006 and 2008, the SHR in Ontario was 380/100,000 person-years which was significantly lower than British Columbia (508/100,000 person-years) and that of the Atlantic region (NB 583/100,000 person-years, NS 633/100,000 person-years, and NL 795/100,000 person-years).

**Table 3:** **SHRs for hospitalizations among kidney transplant patients relative to the Canadian general population, by time since transplantation, 2000 to 2008**^**a**^

**Disease grouping**	**Time since transplantation**							
	**0 days to < 60 days**		**60 days to <3 years**		**3 to <5 years**		**5 years or over**	
	***O***^**b**^	***SHR (95% CI)***	***O***	***SHR (95% CI)***	***O***	***SHR (95% CI)***	***O***	***SHR (95% CI)***
All hospitalizations	3,284	40.1 (38.7, 41.5)	*10,063*	*8.8 (8.7, 9.0)*	2,609	5.5 (5.3, 5.7)	1,475	5.6 (5.3, 5.9)
All hospitalizations excluded ICD-10: S01-T99, V01-Y98, and Z00-Z99^c^	1,371	19.6 (18.6, 20.7)	*7,015*	*7.2 (7.1, 7.4)*	2,044	5.0 (4.8, 5.2)	1,165	5.2 (4.9, 5.5)
Infectious diseases	111	92.5 (76.1, 111.4)	648	38.9 (36.0, 42.0)	124	18.0 (14.9, 21.4)	81	20.8 (16.5, 25.8)
Neoplasms	6	0.8 (0.3, 1.8)	320	3.1 (2.8, 3.5)	105	2.4 (2.0, 2.9)	73	3.0 (2.3, 3.8)
Blood disorders	27	38.6 (25.4, 56.1)	165	17.4 (14.8, 20.2)	24	6.0 (3.8, 8.9)	12	5.5 (2.8, 9.5)
Endocrine disorders	148	67.3 (56.9, 79.0)	773	25.3 (23.5, 27.1)	239	18.5 (16.3, 21.0)	102	14.4 (11.7, 17.4)
Mental disorders	9	2.3 (1.1, 4.4)	43	0.8 (0.6, 1.1)	21	1.0 (0.6, 1.5)	16	1.5 (0.8, 2.4)
Nervous system and sense organs	16	8.4 (4.8, 13.7)	311	11.9 (10.6, 13.3)	112	10.3 (8.5, 12.4)	60	10.0 (7.6, 12.9)
Circulatory	179	14.3 (12.3, 16.6)	633	3.6 (3.3, 3.8)	247	3.2 (2.8, 3.6)	198	4.5 (3.9, 5.2)
Respiratory	31	6.0 (4.0, 8.5)	400	5.4 (4.9, 6.0)	167	5.3 (4.5, 6.2)	94	5.2 (4.2, 6.3)
Digestive	172	17.2 (14.7, 20.0)	1,032	7.5 (7.0, 7.9)	277	4.8 (4.3, 5.4)	171	5.5 (4.7, 6.3)
Skin and subcutaneous tissue	7	7.8 (3.1, 16.0)	103	8.4 (6.9, 10.2)	39	7.8 (5.5, 10.7)	23	8.5 (5.4, 12.8)
Musculoskeletal	18	3.3 (1.9, 5.2)	261	3.4 (3.0, 3.8)	109	3.3 (2.7, 4.0)	50	2.7 (2.0, 3.6)
Genitourinary	411	79.0 (71.6, 87.1)	1,577	21.7 (20.6, 22.8)	375	12.3 (11.1, 13.6)	180	10.6 (9.2, 12.3)
Complications of pregnancy	0	n/a	19	0.2 (0.1, 0.3)	14	0.4 (0.2, 0.6)	5	0.2 (0.1, 0.6)
Congenital anomalies	8	80.0 (34.4, 157.6)	33	22.0 (15.1, 30.9)	13	21.7 (11.5, 37.1)	7	23.3 (9.3, 48.1)
Symptoms ill defined	228	46.5 (40.7, 53.0)	697	10.1 (9.4, 10.9)	178	6.1 (5.2, 7.1)	93	5.7 (4.6, 7.0)

^a^Individuals were followed up from the date of their renal transplant until the date of death or 31 March 2009.

^b^*O* Observed.

^c^Disease category on complications of procedures/transplanted organs rejection, follow-up examination and adjustment, and other supplementary classifications related to transplant procedure (ICD-10 codes: S01-T99, V01-Y98, and Z00-Z99) was excluded.

In Table 3 SHRs of hospital admissions decreased by time since transplantation. In general, the highest SHR was observed in the period immediately after transplant procedure (<60 days); then it decreased and stabilized after 3 years post transplant. Excluding hospitalization <60 days after transplantation disease-specific SHRs were the highest for infectious diseases (SHR=38.9; 95% CI=36.0-42.0) and endocrine disorders (SHR=25.3, 95% CI=23.5-27.1). It is worth noting that the SHRs of infectious diseases and genitourinary diseases were extremely high, but decreased rapidly, while the SHRs for neoplasms and circulatory diseases were relatively stable throughout the follow-up intervals (3.0 and 4.5, respectively), excluding the fellow-up period of <60 days.

**Figure 1:**
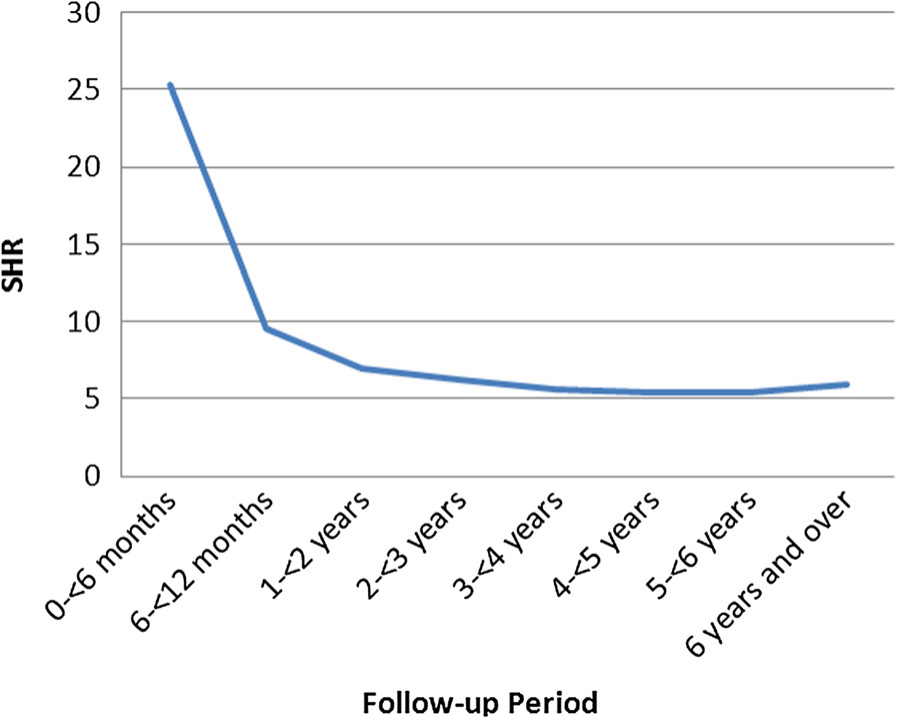
SHRs by follow-up period among kidney transplant patients, 2001 April to 2009 March, Canada.

**Figure 2:**
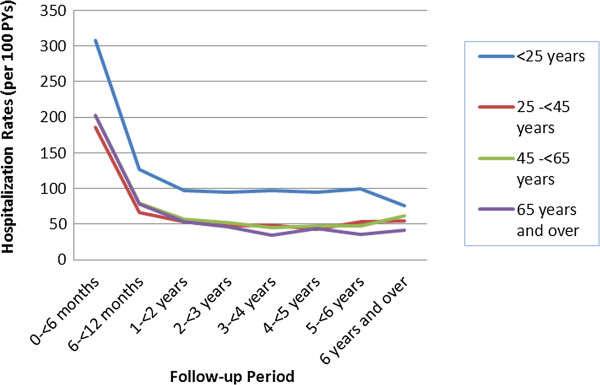
Hospitalization rates by follow-up period among kidney transplant patients, 2001 April to 2009 March, Canada.

Figure 1 displays trends in the hospitalization rates by follow-up period. The highest hospitalization rates were in the first year of follow-up post transplant. After the first year, SHRs decreased considerably for the next 2 to 3 years of follow-up. After 3 to 4 years post transplant, hospitalization rates appeared to level off, and remained stable for the rest of the follow-up period. Figure 2 reveals that such trends were very similar when analyzed separately by age group.

#### ***Morbidity impact on transplant procedure***

Table 4 provided hospital admission rates and SHRs of both pre- (1 April 2001 to 31 March 2003) and post- (1 April 2006 to 31 March 2008) transplant periods among patients who underwent transplantation in 2004. The overall pre-transplant hospital admission rate was 11 times higher than that of the general population while the post-transplant hospital admission rate was 5 times higher. The disease specific statistics shown the SHRs had reduced from 61 to 3 for blood disorders, 57 to 17 for endocrine disorders, 14 to 4 for circulatory diseases, and 43 to 13 for genitourinary diseases. In general, the post-transplant admission rate was about 40% of the pre-transplant hospital rate. With the exceptions of admissions for infection, neoplasms, and respiratory diseases, transplant patients had much lower hospital admission rates post transplantation compared to pre transplantation, especially for blood disorders, endocrine disorders, and circulatory and genitourinary diseases.

**Table 4:** Pre- and post-transplantation hospital admission rates comparison among 728 kidney transplant recipients, 2004

**Disease grouping**	**General population hospital admission rate**	**Pre-transplant hospital admission rate ( *n *) (April 2001 to March 2003)**	**Pre-transplant/ general population**	**Post-transplant hospital admission rate ( *n *) (April 2006 to March 2008)**	**Post-transplant/ general population**	**Post-transplant/Pre-transplant**
All hospitalizations	79.8	1,091.3 (1589)	13.7	436.1 (635)	5.5	0.4
All hospitalizations excluded ICD-10: S01-T99, V01-Y98, and Z00-Z99	68.1	762.4 (1110)	11.2	337.9 (492)	5.0	0.4
Infectious diseases	1.4	15.8 (23)	11.3	20.6 (30)	14.7	1.3
Neoplasms	5.7	16.5 (24)	2.9	22.0 (32)	3.9	1.3
Blood disorders	0.7	42.6 (62)	60.9	2.1 (3)	3.0	0.1
Endocrine disorders	2.0	114.0 (166)	57.0	33.7 (49)	16.9	0.3
Mental disorders	3.9	4.1 (6)	1.1	2.1 (3)	0.5	0.5
Nervous system and sense organs	1.8	30.9 (45)	17.2	15.1 (22)	8.4	0.5
Circulatory	9.7	131.9 (192)	13.6	39.1 (57)	4.0	0.3
Respiratory	6.3	24.7 (36)	3.9	29.5 (43)	4.7	1.2
Digestive	8.8	96.8 (141)	11.0	61.8 (90)	7.0	0.6
Skin and subcutaneous tissue	0.8	7.6 (11)	9.5	4.8 (7)	6.0	0.6
Musculoskeletal	4.5	27.5 (40)	6.1	11.7 (17)	2.6	0.4
Genitourinary	4.7	199.9 (291)	42.5	61.1 (89)	13.0	0.3
Complications of pregnancy	12.1	6.2 (9)	0.5	2.7 (4)	0.2	0.4
Congenital anomalies	0.4	11.7 (17)	29.3	0.7 (1)	1.8	0.1
Symptoms ill defined	4.7	31.6 (46)	6.7	30.9 (45)	6.6	1.0

### Discussion

This study found that kidney transplant recipients had higher hospital admission rates both overall and for many different disease groupings when compared to the general population. These results are consistent across the entire cohort and all the subgroup analyses. A similar observation of higher rates of hospitalization among kidney transplant recipients has been noted by Boubaker et al. who also reported that the most common causes of hospitalization in their study were infections and renal dysfunction [24]. Abbott el al. noted that renal transplant recipients were at high risk for hospitalizations for cytomegalovirus disease and fractures [25,26].

In this patient cohort, the two most common causes of hospitalization were infectious diseases and endocrine disorders. This finding is consistent with other studies [14,24,27,28]. However, over the follow-up period of the study, the hospitalizations for these diseases have decreased greatly. For example, the incidence of infection has dropped from approximately 70% to between 15% and 44% [14]. In our study, except for the first 60 days after a transplant procedure, close to 6% of total hospitalization was associated with infection through the study period. These reductions might be due to recent improvements in surgical techniques, immunosuppressive drugs, methods of diagnosis, and therapy. Urinary tract infection is the most common form of infection, and occurs in approximately 30% of patients within the first 3 months following transplantation [29]. Cytomegalovirus (CMV) is also a common infection among renal transplant patients [26]. The sole exception of an increased SHR was hospitalization due to complications of pregnancy which was likely due a much lower predisposition of transplant patients to become pregnant as these patients are likely older and may already have children or not want to become pregnant. Hospitalizations due to endocrine disorders are likely due to diabetes, a known complication of treatment with steroids. However the data are not granular enough to permit this to be ascertained.

The finding of the highest risk of hospital admission among those follow-up <60 days is not unexpected. This is likely related to the transplant procedure itself in addition to increased intensity of immune suppression during this period. Thereafter, SHR decreased over time, with a stable five-fold increased risk relative to the general population achieved at 3 years post transplant. The differences in hospitalization rates by province in particular between Ontario and BC or Atlantic region may be related to their follow-up care provision after transplant. In BC and Atlantic Canada patients are discharged to the community, and in the rest of the country (including Ontario) most care continues to be provided by the transplant centers.

Given the high quality and completeness in both CORR and DAD, it is reasonable to assume that losses to follow-up and primary diagnostic misclassification were low. As mentioned it has been estimated that <5% of hospital admissions would be missed across Canada (except Quebec), and therefore, the overall bias on the analysis should not be common. It is possible patients would be lost to follow-up if they moved outside the country after transplant procedure. However, given the medical needs of the kidney transplantation patients and the healthcare services provided within Canada, this number would be minimal.

Patients who underwent transplantation in 2004 could provide 2 years of hospital admission data for both pre transplant (2001 to 2003) and post transplant (2006 to 2008), allowing for a comparison of hospital admission between pre and post transplant except transplant procedure. The SHR decreased considerably pre to post transplant, from nearly 11 to approximately 5 suggesting that their overall health status improved significantly.

Caution should be exercised in interpreting the SHRs in this study. First, the DAD is an administrative database which only captures hospitalizations where patients were formally admitted to hospital and only those conditions severe enough to require hospitalization. It is unable to capture the morbidity among kidney transplant patients associated with increased visits to emergency departments where they are treated on an outpatient basis, or increased physician or prescription drug utilization. Second, biases may have been introduced from the patient selection process for transplantation. The selection criterion would differ by individual, and may reflect either a patient with optimum access to or with the most need for a transplant, or in sufficiently good health to be able to undergo transplantation. Since there is no patient level information on the criteria used to identify who underwent transplantation, the magnitude and the direction of the bias are unknown.

## Conclusions

In summary, the present study confirmed and quantified kidney transplant patients’ much higher rates of hospitalization in reference to the general population. The highest cause specific excesses were from infectious diseases and endocrine disorders. Hospital admissions among the patient cohort decreased by time after transplant and reached equilibrium at 3 years. Subsequently, the post-transplant patients had a relative risk for hospitalization approximately 5 times than of the general population. These results may help guide the formulation and implementation of preventive programs after the transplant procedure.

## Abbreviations

CI: Confidence interval; CIHI: Canadian Institute for Health Information; CORR: Canadian Organ Replacement Registry; DAD: Discharge abstract database; ICD: International Classification of Diseases; SHR: Standardized hospitalization ratio.

## Competing interests

The authors declare that they have no competing interests.

## Authors’ contributions

YJ: linked datasets, analyzed data, drafted the manuscript. PV: participated in the data analysis and results interpretation. DS: critically revised the manuscript and helped to finalize the manuscript. YM: designed research and made substantial contributions to interpretation of data. PR: critically revised the manuscript and helped to finalize the manuscript. HM: critically revised the manuscript and gave final approval of the version to be published. All authors read and approved the final manuscript.
